# Polygenic risk score trend and new variants on chromosome 1 are associated with male gout in genome-wide association study

**DOI:** 10.1186/s13075-022-02917-4

**Published:** 2022-10-11

**Authors:** Ya-Sian Chang, Chien-Yu Lin, Ting-Yuan Liu, Chung-Ming Huang, Chin-Chun Chung, Yu-Chia Chen, Fuu-Jen Tsai, Jan-Gowth Chang, Shun-Jen Chang

**Affiliations:** 1 Center for Precision Medicine and Epigenome Research Center, China Medical University Hospital, China Medical University, Taichung, Taiwan; 2 Graduate Institute of Integrated Medicine, College of Medicine, China Medical University, Taichung, Taiwan; 3 Graduate Institute of Clinical Medical Sciences, School of Medicine, China Medical University, Taichung, Taiwan; 4 Division of Laboratory Medicine, China Medical University Hsinchu Hospital, Zhubei City, Taiwan; 5 Division of Immunology and Rheumatology, Department of Internal Medicine, China Medical University Hospital, China Medical University, Taichung, Taiwan; 6 Department of Medical Research, China Medical University Hospital, China Medical University, Taichung, Taiwan; 7 Department of Kinesiology, Health and Leisure Studies, National University of Kaohsiung, No. 700, Kaohsiung University Road, Nanzih District 81148 Kaohsiung, Taiwan

**Keywords:** Gout, Hyperuricemia, Genome-wide association study, Polygenic risk score, Chromosome 1

## Abstract

**Background:**

Gout is a highly hereditary disease, but not all those carrying well-known risk variants have developing gout attack even in hyperuricemia status. We performed a genome-wide association study (GWAS) and polygenic risk score (PRS) analysis to illustrate the new genetic architectures of gout and asymptomatic hyperuricemia (AH).

**Methods:**

GWAS was performed to identify variants associated with gout/AH compared with normouricemia. The participants were males, enrolled from the Taiwan Biobank and China Medical University, and divided into discovery (*n*=39,594) and replication (*n*=891) cohorts for GWAS. For PRS analysis, the discovery cohort was grouped as base (*n*=21,814) and target (*n*=17,780) cohorts, and the score was estimated by grouping the polymorphisms into protective or not for the phenotypes in the base cohort.

**Results:**

The genes *ABCG2* and *SLC2A9* were found as the major genetic factors governing gouty and AH, and even in those carrying the rs2231142 (*ABCG2*) wild-genotype. Surprisingly, variants on chromosome 1, such as rs7546668 (*DNAJC16*), rs10927807 (*AGMAT*), rs9286836 (*NUDT17*), rs4971100 (*TRIM46*), rs4072037 (*MUC1*), and rs2974935 (*MTX1*), showed significant associations with gout in both discovery and replication cohorts (all *p*-values < 1e−8). Concerning the PRS, the rates of gout and AH increased with increased quartile PRS in those SNPs having risk effects on the phenotypes; on the contrary, gout/AH rates decreased with increased quartile PRS in those protective SNPs.

**Conclusions:**

We found new variants on chromosome 1 significantly relating to gout, and PRS predicts the risk of developing gout/AH more robustly based on the SNPs’ effect types on the trait.

**Supplementary Information:**

The online version contains supplementary material available at 10.1186/s13075-022-02917-4.

## Introduction

Gout is known as an inflammatory and complex disease due to the interactions between causal genes and environmental factors. Clustering of hyperuricemia and gout has been known to have familial links, suggesting a high hereditary component to these traits. Mutations have led the genetic variation in modern human populations. Over the past decade, more than 28 SNPs found by genome-wide association study (GWAS) across the world have been shown to relate to gout/hyperuricemia [1–5], but these accounted for only 7% of the variance of in serum urate concentrations [6]. Gouty attack involved both inflammatory and hyperuricemia mechanisms. To identify underlying genetic alterations involved in the two mechanisms, genome-wide association studies (GWASs) have been conducted in many populations, and genetic loci covering *ABCG2* and *SLC2A9* have been found most repeatedly and significantly [7–11].

Most of the genes identified in current GWASs seem plausible contributors to serum uric acid levels because they encode for proteins that are responsible for uric acid excretion in the intestines or kidney, including the genes *ABCG2* and *SLC2A9* [12, 13]. However, except for rs2231142 in *ABCG2*, which is a missense variant, most genetic variants that have been reported to be significant so far are located in intronic or intergenic regions, and causative variants have not been identified for most loci.

Not all those carrying genetic variants of the aforementioned genes would developing gout disease. This may involve unknown genes or the mechanism of genetic interaction. However, gout attack is a complex disease, and an estimation of polygenic risk score (PRS) is one way by which people can measure their risk of developing clinical diseases [14, 15]. PRS based on the total number of changes related to the disease is an estimate of an individual’s genetic liability to a trait, calculated according to their genotype profile and relevant genome-wide association study (GWAS) data. Here, we performed a GWAS with a large sample of male participants to reveal the genetic contributions and estimation of PRS on the phenotypes of gout and asymptomatic hyperuricemia (AH).

## Methods

### Study population

We aimed to explore the genetic variants associated with the development of gout and AH and enrolled 39,594 male participants as a discovery cohort. A second cohort with whole genome sequence data was included as a replication cohort, which was composed of 891 male participants. All participants were aged between 30 and 70 years old, and they were selected from Taiwan Biobank and China Medical University in Taiwan. We grouped the participants of the discovery cohort into base cohort (*n*=21,814) and target cohort (*n*=17,780) for PRS analysis (Supplementary Table 1). PRSice-2 software (version 2.3.3 for R) was applied for the estimation of PRS with applying the odds ratios (OR) estimated by GWAS data in the base cohort [16], which was adjusted by age, and then we estimated the individual PRS of gout/AH phenotype for the target cohort. The threshold for p was set to 5e−5 in the PRS analysis, which can ensure to retrieve enough significant variants for estimating individual PRS. In order to explore the genetic component without confounded by rs2231142 (*ABCG2* gene) which was the most significant variant with gout in Taiwan [17], we further analyzed the associations between variants and gout/AH under a selection of those carrying rs2231142 wild-type (GG). TWB is a nationwide database for research, which combines genomic profiles with lifestyle patterns from people in Taiwan to explore the relationships between diseases and genetics. Each participant underwent biochemical testing (with blood samples) and physical examination; all of them also signed informed consent for genotyping analysis. The phenotype of gout disease was self-reported and previous studies utilizing a similar method of self-reported gout suggested that self-reporting of physician-diagnosed gout retained good reliability and sensitivity [18, 19]. Those with serum uric acid more than or equal to 7 mg/dl without gout history were defined as AH cases.

The ethics committee of the China Medical University Hospital Institutional Review Board in Taiwan (CMUH108-REC1-091) approved this project. Both the Declaration of Helsinki and the Good Clinical Practice Guidelines were followed and informed consent was written by all participants.

### Genotyping and quality control

The participants of the discovery cohort were genotyped using Affymetrix Axiom genotyping array (chip: TWB2), including 680K SNPs. Quality control (QC) was applied to leave out those SNPs with low call rate (< 95%), minor allele frequency (MAF) less than 0.01, and deviation from the Hardy-Weinberg equilibrium (*p* < 0.0001; library HardyWeinberg of R program). A total of 401,037 SNPs passed the quality control for GWAS. The participants of replicated cohort underwent whole genome sequencing on the Illumina Hiseq platform. For the Illumina Hiseq platform, variant calling was performed with Isaac Variant Caller version 2.0.17, Grouper version 1.4.2, and CNVseg version 2.2.4. Alleles were annotated with ANNOVAR [20]. Some of the significant polymorphisms found by the discovery cohort were extracted from the variants of variant call format (VCF). All the genetic positions displayed in this study were GRCh38 format.

### Statistical analysis

The clinical characteristics of subjects with and without gout and AH were compared by applying a chi-square test for categorical variables and *t*-test or ANOVA for continuous variables. The *p*-values estimated by chi-square test (chisq.test function of R program) for the association between genetic variants located on each chromosome for the gout/AH were presented in Manhattan plots (library qqman) by using the R-program provided by Turner [21]. We conducted a logistic regression model for estimating the odds ratios (ORs) as well as 95% confidence intervals (95% CI) for the associations between genetic variants and gout/AH after adjustment of age (library aod of R program). The Locuszoom plot was employed to visualize the regional strength and associations between loci and gout/hyperuricemia related to local linkage-disequilibrium (LD), and genomic position (locuszoom v1.4) [22]. A heatmap of paired LD for significant SNPs related to gout/AH was displayed for the variants located in the same chromosome (LDheatmap library) [23]. We used PLINK (v1.9), PERL (v5.16), and R (v3.6) programs (CentOS v7.0) to mine raw data, estimate *p*-values, and draw the figures. For the GWAS, after Bonferroni correction for multiple testing, the significance was determined at *p* < 1 × 10^−8^. For other analyses, significance was determined at *p* < 0.05.

## Results

A total of 39,594 male participants were included as the discovery cohort in this study, which was composed of 5857 gout patients, 12,382 AH subjects, and 21,355 normouricemia participants. The mean age of the three groups were 54.00 (± 10.65), 51.27 (± 11.30), and 51.72 (± 11.04) years old, and the mean uric acid levels were 8.79 (± 2.26 mg/dl), 8.09 (± 1.12 mg/dl), and 5.72 (±0.86 mg/dl), respectively (Supplementary Table 1).

**Fig. 1:**
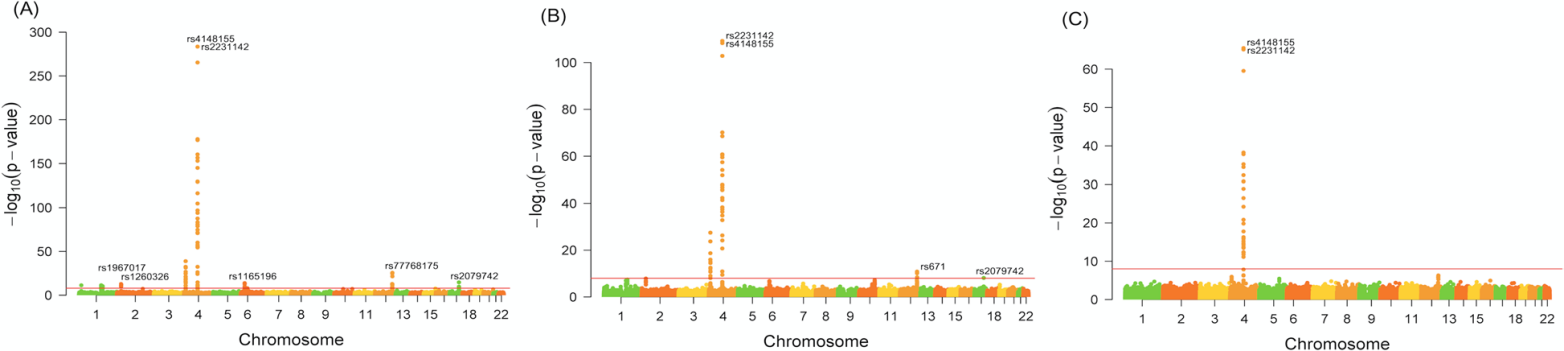
The Manhattan plots reveal the *p*-values of variants related to gout and asymptomatic hyperuricemia (AH). The *p*-values were estimated for developing gout compared to normal (**A**). When compared to normal, the *p*-values of variants related to AH are illustrated (**B**), and compared to AH, the values of variants related to gout are illustrated (**C**). The red horizontal line is denoted as a cut-off for the *p*-value of significant difference between the phenotypes and variants by 1e−8

To explore the genetic associations of SNPs with the phenotypes, we illustrated the Manhattan plots which showed all *p*-values of SNPs related to gout/AH across 23 pairs of chromosomes, and the result showed a total of 120 variants significantly associated with gout, located in a total of 35 genes (Supplementary Table 2; Fig. 1A). The genes which contain more than or equal to two significant SNPs include genes *ABCG2*, *SLC2A9*, *SLC17A1*, *ZNF518B*, *SLC17A3*, *GCKR*, *PKD2*, *MEPE*, *PDZK1*, *HECTD4*, *CD160*, *DNAJC16*, *TRIM46*, *CCDC63*, *ALDH2*, and *CUX2*. Concerning AH, a total of 57 SNPs located in 13 genes were significantly associated with hyperuricemia compared to normal subjects (Supplementary Table 3; Fig. 1B). The genes of the variants were located in *ABCG2*, *SLC2A9*, *ZNF518B*, *PKD2*, *MEPE*, *ALDH2*, *HECTD4*, *BRAP*, *SPP1*, *MAPKAPK5*, *CLNK*, *ACAD10*, and *BCAS3*. A total of 27 SNPs were significantly related to gout compared to AH, located in genes *ABCG2*, *PKD2*, and *SPP1* (Supplementary Table 4; Fig. 1C). Interestingly, *ABCG2*, *PKD2*, and *SPP1* genes were found to be significantly related to gout/AH in all three comparisons (gout vs normal; gout vs AH; AH vs normal).

**Fig. 2:**
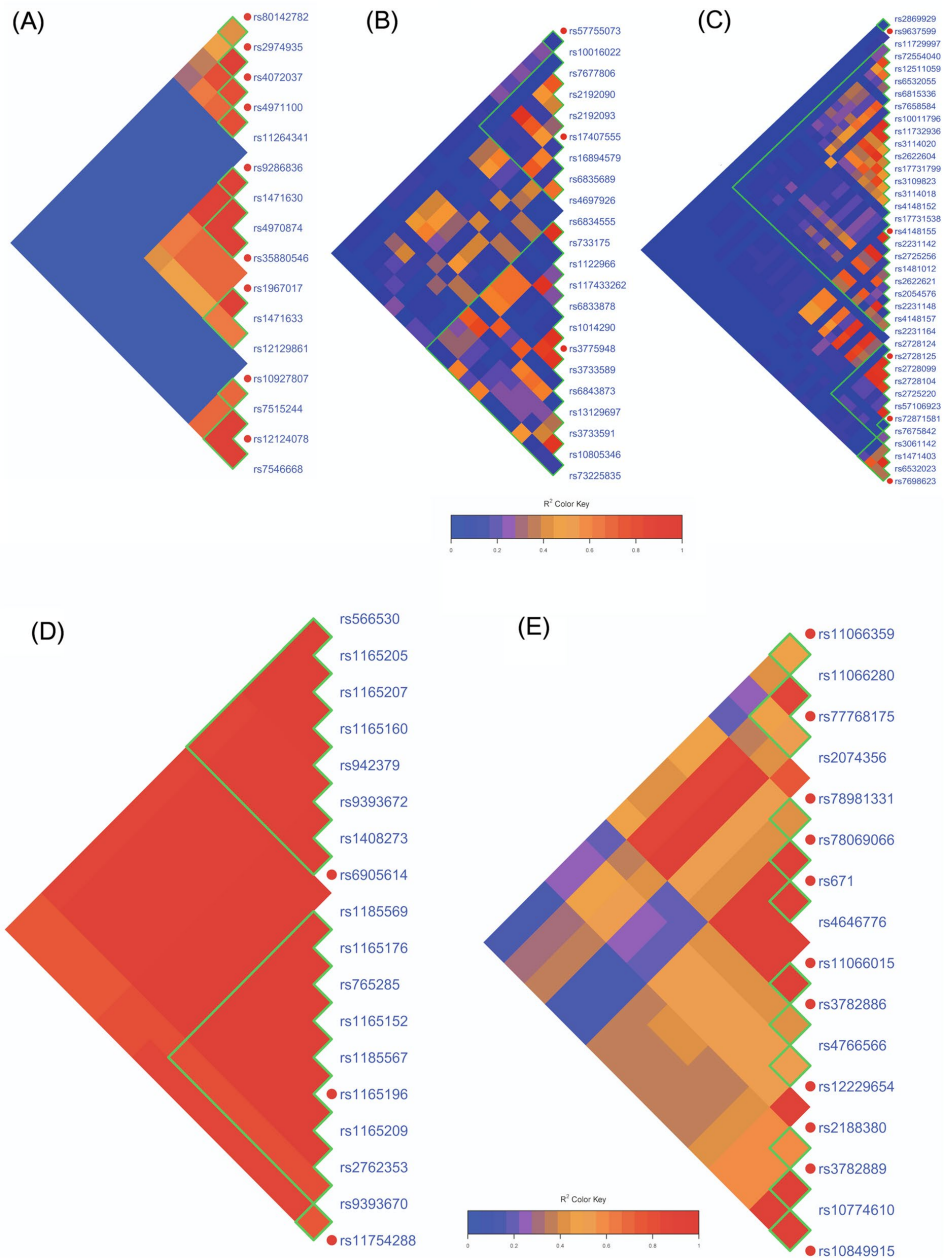
The paired linkage disequilibrium (LD) of the variants related to gout. The LD illustrates the variants of developing gout compared to normal in chromosome 1 (**A**), chromosome 4 on p arm (**B**), chromosome 4 on q arm (**C**), chromosome 6 (**D**), and chromosome 12 (**E**). The green line blocks the gene, and the red circle indicates it is the most significant variant in its gene

Regarding LD between those significant variants, we applied a heatmap of paired variants to show the significant variants of LD degree for gout/AH (Fig. 2). Three LD blocks existed in chromosome 1 for the variants relating to gout (Fig. 2A), which were composed of genes *DNAJC16*, *AGMAT* (first block), *PDZK1*, *CD160*, *NUDT17* (second block), *TRIM46*, *MUC1*, *MTX1*, and *ASH1L* (third block). Surprisingly, the results showed that three variants of *DNAJC16* and one variant of *AGMAT* were significantly associated with gout, which had not been found before, namely, rs7546668 (*DNAJC16*), rs12124078 (*DNAJC16*), rs7515244 (*DNAJC16*), and rs10927807 (*AGMAT*). Furthermore, the four polymorphisms existed in a high LD degree (Supplementary Fig. 1). Two major LD blocks relating to gout were observed in chromosome 4 which is located on the p arm and q arm (Fig. 2B, C); the major causal genes are *SLC2A9* and *ABCG2*, respectively. Moreover, all the variants in chromosomes 6 (Fig. 2D) and 12 (Fig. 2E) existed in high LD in each chromosome. Concerning AH, the result also revealed two major blocks and one block associated with hyperuricemia in chromosomes 4 and 12, respectively (Supplementary Fig. 2).

To explore causal variants without the confounding effect caused by the variant rs2231142, we further estimated the associations between the SNPs and the gout/AH phenotypes under the selection limitation of those carrying wild genotypes (GG) of rs2231142 (*n*=18,177). We applied Manhattan plots to show the significant variants across all chromosomes (Supplementary Fig. 3). The results showed 12 variants were significantly related to gout compared to normal, four variants significantly related to AH compared to normal, and none was found to be significantly related to gout compared to AH (Supplementary Table 5). The involved genes were *ABCG2*, *SLC2A9*, *PKD2*, *ZNF518B*, *ALDH2*, *HECTD4*, and *MAPKAPK5* in the phenotype of gout; and only gene *SLC2A9* was found in the AH phenotype. Furthermore, we estimated the OR of developing the phenotypes of the abovementioned 16 variants which were significantly related to the phenotypes under the wild-type selection of rs2231142 (Table 1). The results revealed that the variants exhibited protective effects against developing gout, namely rs3733589 (*SLC2A9*), rs3775948 (*SLC2A9*), rs1014290 (*SLC2A9*), rs3109823 (*ABCG2*), rs2622604 (*ABCG2*), rs6532055 (*ABCG2*), rs72554040 (*ABCG2*), rs671 (*ALDH2*), rs78069066 (*MAPKAPK5*), and rs77768175 (*HECTD4*). Polymorphisms rs17407555 (*ZNF518B*) and rs2725231 (*PKD2*) showed risk effects, however. The polymorphisms located in chromosome 12 showed protective effects, and all existed in a high LD (Supplementary Fig. 4). Concerning the development of AH, the polymorphism rs10805346 (*SLC2A9*) acted as a risk factor, but rs3733589 (*SLC2A9*), rs3775948 (*SLC2A9*), and rs1014290 (*SLC2A9*) acted as protective variants for the phenotype (Table 1), and these existed in a weak LD (*r*^2^ >0.22; Supplementary Fig. 5).

**Table 1:** The OR and 95% CI of variants associating with gout/hyperuricemia in those carrying rs2231142 (*ABCG2*) wild type (GG)

No.	SNP	Gout/hyperuricemia	Normal	OR (95% CI)	*p*-values
**Relationship between gout and normal subjects (** ***n*** **=13,088)**					
1	rs3733589 (SLC2A9; chr4:9985700)				
	GG	754 (42.38)	4019 (35.62)		
	GA	820 (46.09)	5393 (47.80)	0.81 (0.73-0.90)	1.16e-04
	AA	205 (11.52)	1870 (16.58)	0.58 (0.50-0.69)	8.90e-11
2	rs3775948 (SLC2A9; Chr4:9993558)				
	GG	719 (40.39)	3721 (33.07)		
	GC	835 (46.91)	5386 (47.87)	0.80 (0.72-0.89)	6.40e-05
	CC	226 (12.70)	2144 (19.06)	0.55 (0.47-0.64)	3.74e-14
3	rs1014290 (SLC2A9; Chr4:10000237)				
	GG	736 (41.30)	3900 (34.53)		
	GA	838 (47.03)	5376 (47.60)	0.83 (0.74-0.92)	4.70e-04
	AA	208 (11.67)	2019 (17.88)	0.55 (0.46-0.64)	1.85e-13
4	rs17407555 (ZNF518B; chr4:10273370)				
	AA	1155 (64.85)	8146 (72.11)		
	AG	563 (31.61)	2896 (25.64)	1.37 (1.23-1.53)	1.38e-08
	GG	63 (3.54)	255 (2.26)	1.74 (1.31-2.31)	9.67e-05
5	rs2725231 (PKD2; chr4:88013803)				
	AA	694 (39.01)	5138 (45.52)		
	AG	835 (46.94)	5011 (44.39)	1.23 (1.11-1.37)	1.34e-04
	GG	250 (14.05)	1139 (10.09)	1.63 (1.39-1.90)	1.37e-09
6	rs3109823 (ABCG2; chr4:88143450)				
	CC	942 (52.95)	5154 (45.71)		
	CT	711 (39.97)	4954 (43.93)	0.79 (0.71-0.87)	6.06e-06
	TT	126 (7.08)	1168 (10.36)	0.59 (0.48-0.72)	1.09e-07
7	rs2622604 (ABCG2; chr4:88157772)				
	TT	1017 (57.10	5388 (47.72)		
	TC	654 (36.72)	4875 (43.18)	0.71 (0.64-0.79)	2.06e-10
	CC	110 (6.18)	1027 (9.10)	0.57 (0.46-0.70)	6.43e-08
8	rs6532055 (ABCG2; chr4:88197235)				
	TT	869 (48.93)	4621 (41.00)		
	TC	729 (41.05)	5202 (46.15)	0.75 (0.67-0.83)	5.20e-08
	CC	178 (10.02)	1448 (12.85)	0.65 (0.55-0.78)	1.06e-06
9	rs72554040 (ABCG2; chr4:88231172)				
	GG	544 (30.60)	2644 (23.40)		
	GA	824 (46.34)	5581 (49.40)	0.72 (0.64-0.81)	3.03e-08
	AA	410 (23.06)	3072 (27.19)	0.65 (0.56-0.74)	7.15e-10
10	rs671 (ALDH2; chr12:111803962)				
	GG	1031 (57.89)	5656 (50.06)		
	GA	624 (35.04)	4695 (41.55)	0.73 (0.66-0.81)	5.89e-09
	AA	126 (7.07)	948 (8.39)	0.73 (0.60-0.89)	1.64e-03
11	rs78069066 (MAPKAPK5; chr12:111900120)				
	GG	1020 (57.43)	5601 (49.68)		
	GA	630 (35.47)	4706 (41.74)	0.74 (0.66-0.82)	1.41e-08
	AA	126 (7.09)	967 (8.58)	0.72 (0.59-0.87)	8.40e-04
12	rs77768175 (HECTD4; chr12:112298314)				
	0AA	1029 (57.87)	5641 (50.05)		
	1AG	625 (35.15)	4688 (41.60)	0.73 (0.66-0.81)	7.66e-09
	2GG	124 (6.97)	941 (8.35)	0.72 (0.59-0.88)	1.28e-03
**Relationship between hyperuricemia and normal subjects (** ***n*** **=16,395)**					
1	rs10805346 (SLC2A9; chr4:9918723)				
	TT	2302 (45.28)	5677 (50.28)		
	TC	2251 (44.28)	4626 (40.97)	1.20 (1.12-1.29)	3.11e-07
	CC	531 (10.44)	988 (8.75)	1.33 (1.18-1.49)	1.86e-06
2	rs3733589 (SLC2A9; chr4:9985700)				
	GG	2005 (39.45)	4019 (35.62)		
	GA	2441 (48.02)	5393 (47.80)	0.91 (0.84-0.97)	7.93e-03
	AA	637 (12.53)	1870 (16.58)	0.68 (0.62-0.76)	7.77e-13
3	rs3775948 (SLC2A9, Chr4:9993558)				
	GG	1886 (37.18)	3721 (33.07)		
	GC	2490 (49.08)	5386 (47.87)	0.91 (0.85-0.98)	1.34e-02
	CC	697 (13.74)	2144 (19.06)	0.64 (0.58-0.71)	9.57e-18
4	rs1014290 (SLC2A9; chr4:10000237)				
	GG	1957 (38.49)	3900 (34.53)		
	GA	2464 (48.46)	5376 (47.60)	0.91 (0.85-0.98)	1.40e-02
	AA	664 (13.06)	2019 (17.88)	0.64 (0.57-0.71)	1.56e-17

*OR* odds ratio, *95% CI* 95% confidence intervals

The replication cohort revealed significant variants of chromosome 1 which were found in the discovery cohort. The results showed genotype TT of rs10927807 located in gene *AGMAT* revealed 4.34 times risk for developing gout (OR=4.34, 95% CI =2.68–7.02, *p*=6.62e−10; Table 2). Moreover, rs9286836 (*NUDT17*) and rs4971100 (*TRIM46*), rs4072037 (*MUC1*) and rs2974935 (*MTX1*) also revealed significant associations with gout (all *p*-values <1e−8).

**Table 2:** The associations between gout and significant variants of chromosome 1 in replication cohort

SNP (chr; position; gene)	Gout	Control	OR (95% CI)	*p*-values
rs7546668 (chr1; 15528628; DNAJC16)				
GG	55 (27.0)	89 (18.8)	2.77 (1.69-4.56)	4.31e-05
GC	114 (55.9)	227 (48.0)	2.25 (1.47 -3.46)	1.74e-04
CC	35 (17.2)	157 (33.2)	1.0	
rs12124078 (chr1; 15543404; DNAJC16)				
AA	55 (26.8)	90 (19.0)	2.60 (1.58-4.26)	1.22e-04
AG	114 (55.6)	230 (48.6)	2.11 (1.37-3.23)	5.38e-04
GG	36 (17.6)	153 (32.3)	1.0	
rs7515244 (chr1;15546891; DNAJC16)				
AA	55 (26.8)	91 (19.2)	2.58 (1.57-4.21)	1.45e-04
AG	114 (55.6)	229 (48.4)	2.12 (1.38-3.24)	4.99e-04
GG	36 (17.6)	153 (32.3)	1.0	
rs10927807 (chr1; 15584515; AGMAT)				
TT	66 (32.4)	74 (15.6)	4.34 (2.68-7.02)	6.62e-10
TG	100 (49.0)	214 (45.2)	2.28 (1.49-3.47)	1.09e-04
GG	38 (18.6)	185 (39.1)	1.0	
**rs12129861 (chr:1; 145709377; PDZK1)**				
**TT**	**10 (4.90)**	**31 (6.55)**	**0.62 (0.30 -1.31)**	**0.206**
**CT**	**56 (27.45)**	**176 (37.21)**	**0.61 (0.43-0.88)**	**0.008**
**CC**	**138 (67.65)**	**266 (56.24)**	**1.0**	
**rs1471633 (chr:1; 145711327; PDZK1)**				
**GG**	**10 (4.88)**	**23 (4.86)**	**0.86 (0.40 -1.86)**	**0.708**
**TG**	**50 (24.39)**	**162(34.25)**	**0.61 (0.42 -0.89)**	**0.010**
**TT**	**145 (70.73)**	**288(60.89)**	**1.0**	
rs1967017 (chr:1;145711421; PDZK1)				
GG	10(4.88)	23(4.86)	0.86 (0.40-1.86)	0.708
AG	50(24.39)	162(34.25)	0.61 (0.42-0.89)	0.010
AA	145(70.73)	288(60.89)	1.0	
rs35880546 (chr:1;145722014; CD160)				
GG	8(3.90)	15(3.17)	1.07 (0.44-2.57)	0.886
AG	44(21.46)	152(32.14)	0.58 (0.39-0.85)	0.005
AA	153(74.63)	306(64.69)	1.0	
rs4970874 (chr:1;145725691; CD160)				
GG	8(3.90)	15(3.17)	1.07 (0.44-2.57)	0.886
AG	44(21.46)	152(32.14)	0.58 (0.39-0.85)	0.005
AA	153(74.63)	306(64.69)	1.0	
rs1471630 (chr:1;145727789; CD160)				
CC	8(3.90)	15(3.17)	1.07 (0.44-2.57)	0.886
TC	44(21.46)	152(32.14)	0.58 (0.39-0.85)	0.005
TT	153(74.63)	306(64.69)	1.0	
rs9286836 (chr:1;145846532; NUDT17)				
A/A	110(53.66)	17(3.59)	39.75 (21.96-71.94)	5.47e-50
A/C,G	46(22.44)	155(32.77)	1.82 (1.17-2.85)	0.008
C,G/C,G	49(23.90)	301(63.64)	1.0	
rs11264341 (chr:1;155179017; TRIM46)				
CC	68(33.17)	47(9.94)	7.12 (4.39-11.53)	2.77e-17
CT	88(42.93)	185(39.11)	2.34 (1.57-3.48)	2.24e-05
TT	49(23.90)	241(50.95)	1.0	
rs4971100 (chr:1;155183255; TRIM46)				
GG	74(36.27)	39(8.25)	10.53 (6.43-17.24)	3.35e-24
GA	81(39.71)	162(34.25)	2.78 (1.85-4.16)	4.52e-07
AA	49(24.02)	272(57.51)	1.0	
rs4072037 (chr:1;155192276; MUC1)				
CC	85(41.46)	29(6.13)	17.13 (10.23-28.68)	2.87e-34
CT	69(33.66)	146(30.87)	2.76 (1.83-4.17)	8.37e-07
TT	51(24.88)	298(63.00)	1.0	
rs2974935 (chr:1;155212052; MTX1)				
GG	86(42.16)	28(5.92)	17.67 (10.52-29.65)	1.05e-34
GT	66(32.35)	146(30.87)	2.60 (1.72-3.93)	4.05e-06
TT	52(25.49)	299(63.21)	1.0	
rs80142782 (chr:1;155515236; ASH1L)				
CC	2(0.98)	9(1.90)	0.55 (0.12-2.57)	0.439
TC	53(25.85)	94(19.87)	1.39 (0.95-2.05)	0.094
TT	150(73.17)	370(78.22)	1.0	

*Chr* chromosome

**Fig. 3:**
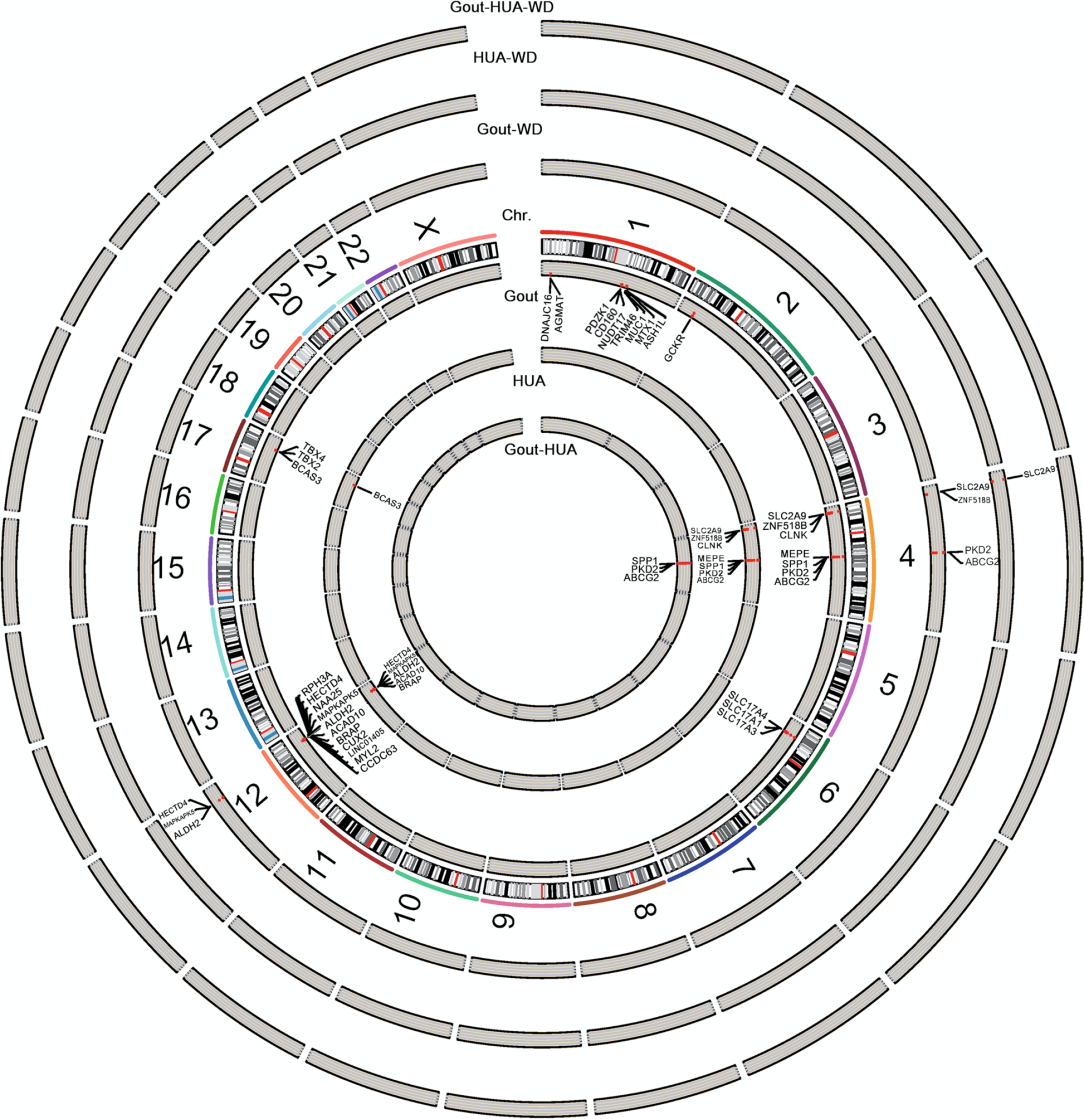
The circular map illustrates the genes and locations of significant variants related to the phenotypes. Gout: the comparison between gout and normal; Gout-WD: the same comparison under a selection of those carrying rs2231142 wild-genotype GG. HUA: the comparison between hyperuricemia and normal; HUA-WD: the same comparison under a selection of those carrying rs2231142 wild-genotype GG. Gout-HUA: the comparison between gout and hyperuricemia; Gout-HUA-WD: the same comparison under a selection of those carrying rs2231142 wild-genotype GG

A summary circle figure illustrates the aforementioned six phenotypes to exhibit the genes with significant variants (Fig. 3). The results showed *ABCG2* gene was involved in four phenotypes, except for those developing hyperuricemia and gout (compared to AH) under the selection of rs2231142 wild type. Notably, the *SLC2A9* exhibited effects on gout and AH phenotypes under a selection of those carrying rs2231142 wild-genotype, but the effect was not observed in developing gout compared to AH.

**Fig. 4:**
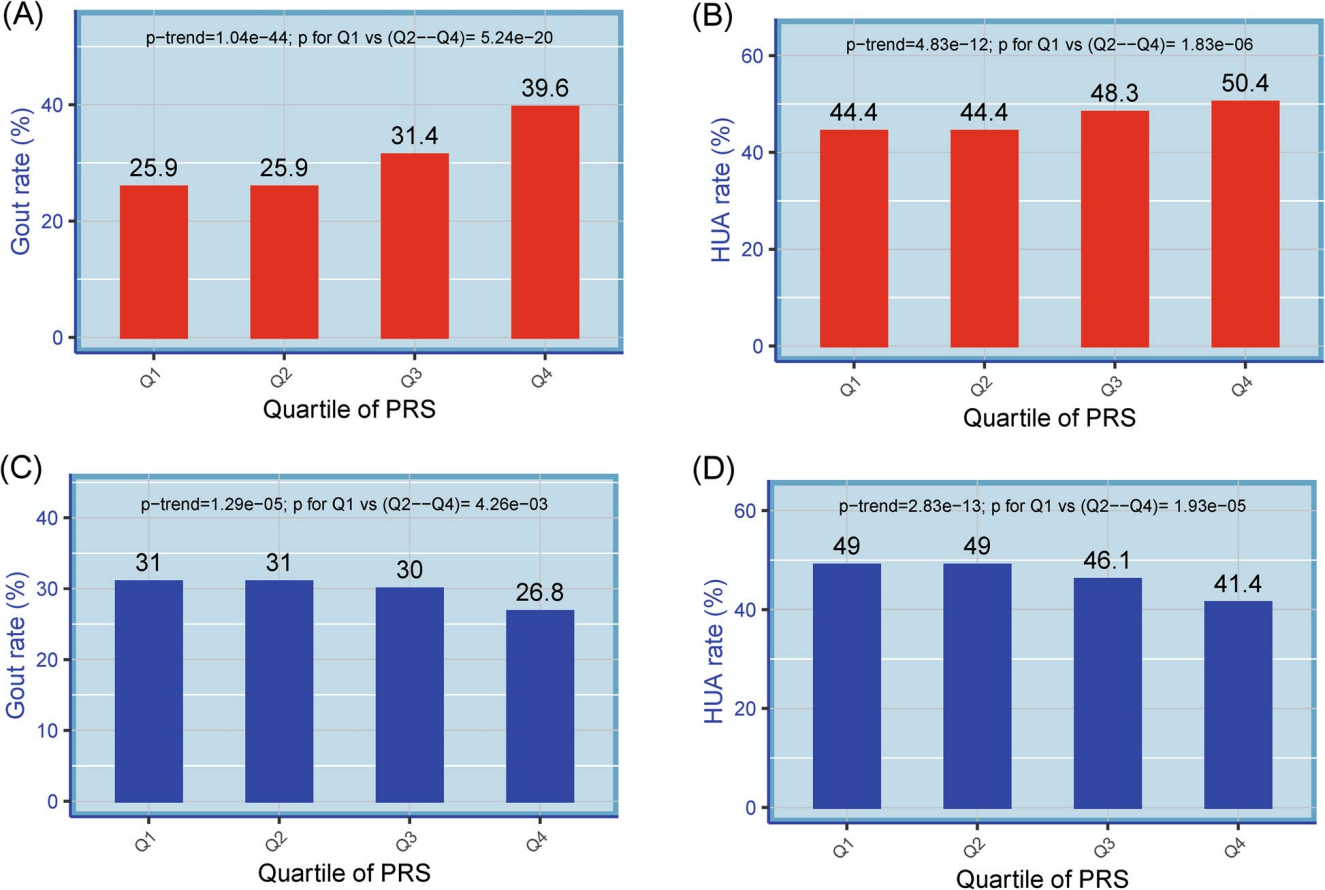
The rates of gout and asymptomatic hyperuricemia (AH) compared to those with normouricemia among different quartile polygenic risk scores (PRS). The rates of gout and AH increased with increased quartile PRS in those variants with risk effects (odds ratio (OR) >=1; red color) of developing the phenotypes (**A**, **B**, respectively). Whereas the rates of gout and AH decreased with increased quartile PRS in those variants with protective effects (OR <1; blue color) of developing the phenotypes (**C**, **D**, respectively). The rates of gout/AH were calculated only in the participants for estimating PRS, but not in the whole population. HUA, hyperuricemia

Concerning the associations between PRS and gout/AH phenotypes, compared to the controls. More than 13 significant variants were retrieved from each PRS analysis. The rates of gout and AH increased with increased quartile PRS in those variants showing risk effects on the phenotypes (all *p*-trend < 1e−11; Fig. 4A, B). On the contrary, for those variants with protective effects on gout/AH, the trend decreased with increased quartile PRS (all *p*-trend < 1e−4; Fig. 4C, D). Interestingly, these rates of gout/AH in Q4 of PRS also showed significant association with the rates in other three quartiles regardless of whether the variants had risk effects or not (all *p*-values < 0.01).

## Discussion

We performed a large GWAS to explore the genetic variants and PRS relating to gout and AH in males, and we found that the variants located in genes *ABCG2* and *SLC2A9* were the major genetic factors governing gouty attack and hyperuricemia. Surprisingly, variants on chromosome 1 located in genes *DNAJC16*, *AGMAT, NUDT17, TRIM46, MUC1* and *MTX1* showed significant associations with gout, which finding had not been reported before. Genes *AGMAT* and *DNAJC16* exist a high LD. Recently, Yamamoto et al. revealed *DNACJ16* to be involved in the autophagy process delivering the engulfed substrates into lysosomes for degradation**;** moreover, the same researchers also reviewed recent advances in the relationship between autophagosome formation and endoplasmic reticulum [24], and they showed that *DNAJC16* overexpression extended the size of the autophagosome through its DnaJ and TRX domains and that *DNAJC16* ablation resulted in a defect in engulfing larger targets [25]. The phagocytosis of monosodium urate crystals triggering an inflammatory cellular state in synoviocytes suggests a possible mechanism in the pathogenesis of crystal-induced arthritis. [26] In a previous study, Chen et al. explored a cell model to show significantly higher IL-8 release from endothelial cells combined with *ABCG2* knockdown [17]. Liao et al. found that monosodium urate (MSU) alone induced *cGKII* and *TLR2* expression with increased phagocytosis activity, which suggested that cGKII knockdown significantly inhibited this MSU-induced cGKII-TLR2-phagocytosis axis [27]. Herein, we suggest that the new gene *DNAJC16* associated with gout disease may be involved in the mechanism of urate phagocytosis.

Arginase is found ubiquitously in mammals, and there are two distinct isoforms. Isoform-I is expressed predominantly in the liver and plays an important role in the urea cycle; its reaction product, ornithine, is a precursor for the synthesis of the amino acid proline and glutamic acid for cellular replication [28]. Isoform-II is widely expressed in mitochondria in the extrahepatic tissues and is believed to play an important role in endothelial dysfunction [29]. Interestingly, Arginase is also involved in the control of nitric oxide synthesis, and its function is associated with hypertension, inflammation and diabetes [29, 30]. *AGMAT* encodes agmatinase, which is a metallohydrolase involved in the hydrolysis of agmatine to produce urea and putrescine. This enzyme plays a critical role in polyamine biosynthesis and the regulation of agmatine levels. Early evidence showed LPS induced a dose-dependent stimulation of agmatinase, and exposure to IL-10 and TGF-beta caused a reduction in agmatinase and arginine decarboxylase (ADC), whereas IL-4 was ineffective on ADC, but reversed the LPS-induced increase of agmatinase [31], which suggests a regulatory role of agmatine during inflammation. Sastre et al. discovered that both ADC and agmatinase are constitutively expressed in macrophages [31], with activities comparable to those reported in the brain or kidney [32, 33]. In addition to its role as a component of proteins, arginine is a precursor for the synthesis of other molecules including nitric oxide (NO), citrulline, agmatine, urea, and ornithine. Two well-described pathways of L-arginine metabolism in inflammation are the conversion of arginine to NO and citrulline by NOS and the breakdown of arginine to urea and ornithine by arginase. These pathways are temporally regulated in inflammatory models including wound healing and glomerulonephritis [34–36]. Although we found a higher association between gout and *AGMAT* than between gout and *DNACJ16* in the replication cohort, from the above literature review, both genes may possibly cause gout.

Even under selection limitation of those carrying rs2231142 wild genotype in this study, the major risk genes for developing gout were located in chromosomes 4 and 12, including region of chromosome 4 of *SLC2A9*, *ZNF518B*, *PKD2*, *ABCG2*, and the region of chromosome 12 of genes *ALDH2*, *MAPKAPK5*, and *HECTD4*. This illustrates that these genes show effects on gout independently of rs2231142 (*ABCG2*). Moreover, the variants of *SLC2A9* show protective and opposing effects on hyperuricemia in those carrying the rs2231142 wild genotype. The *SLC2A9* gene was shown to associate with lower uric acid levels and hypouricemia. The effect of contributing to uric acid was more pronounced in those without carrying the rs2231142 variant.

*SLC2A9* encodes a transporter protein belonging to class II of the facilitative glucose transporter family (GLUT). GLUT9 is expressed in the proximal tubules [37], and it mediates sodium-independent hexose uptake into target cells by facilitated diffusion [38]. Fructose is potentially a substrate of GLUT9, and it was identified as a marker for uric acid concentrations [39]. Compared with other carbohydrates, fructose is associated with higher levels of uric acid, triglycerides, and cholesterol [40]. Sex-specific differences in the effects of *SLC2A9* have also been observed in previous studies. Doring et al. found that the variance of uric acid levels explained by *SLC2A9* was five-fold higher in females than in males, and the percentage of variance accounted for by *SLC2A9* expression levels was 3.5% in men and 15% in women [41].

*ABCG2* is a urate transporter protein that regulates uric acid excretion in the kidney, intestine, and liver [42–44]. Woodward et al. discovered that ABCG2 is located at the apical border membrane of renal proximal tubule cells and plays a role in uric acid secretion [45]. Furthermore, the Q141K mutation encoded by rs2231142 resulted in 53% lower urate transport rates compared to the wild type. Moreover, another pathway for uric acid elimination was recently discovered in the intestine. In a nephrectomized rat model, *ABCG2* was significantly upregulated in the ileum compared to wild-type rats [13]. Moreover, Merino et al. found that expression of the hepatic protein BCRP1, a substrate of *ABCG2*, is higher in male mice than in female mice; they also found that hepatic expression of human BCRP/ABCG2 was higher in men [46].

Although Hunter and Mars et al. suggested a PRS model which, by adding up the number of risk variants, could be applied to estimate the genetic risk of gene-susceptible diseases [47, 48]. Our data revealed that the rates of gout and AH increased with quartile of PRS in the variants of showing risk effects on the phenotypes, while the rates decreased with increased quartile PRS in the protective variants.

## Conclusions

This study performed a GWAS in a large sample size and found genes *DNAJC16*, *AGMAT*, *NUDT17*, *TRIM46 MUC1*, and *MTX1* on chromosome 1 relating to gout disease. Gene *SLC2A9* is the major independent gene of controlling uric acid and governing gout attack in those carrying rs2231142 wild-type; and PRS provides a robust insight into the genetic component of gout and AH in the selection of variant-effect types on the traits.

## Supplementary Information

**Additional file 1:** **Supplementary Table1.** The distribution of age and uric acid of those participated in the studycohort.

**Additional file 2:** **Supplementary Table 2.** The susceptible variants significantlyassociated with gout compared to normal.

**Additional file 3:** **Supplementary Table 3.** The susceptible variants significantly associated with hyperuricemia comparedto normal.

**Additional file 4:** **Supplementary Table 4.** The susceptible variants significantly associated with gout compared tohyperuricemia. 

**Additional file 5:** **Supplementary Table5.** The susceptible variants of significantly associated with gout andhyperuricemia in those with wild genotype of top significant variants(rs2231142).

**Additional file 6:** **SupplementaryFigure 1.** The linkage disequilibrium of the four variants significantlyassociated with gout in genes DNAJC16 and AGMAT. The variant rs7546668 showedhigh associations with the other three variants (*r*^2^ >=0.68). Thered line indicates the cut-off significant p-value by 1e-8.

**Additional file 7:** **Supplementary Figure 2.** The paired linkagedisequilibrium among the variants which were significantly associated withhyperuricemia on chromosome 4 (A) and 12 (B).

**Additional file 8:** **SupplementaryFigure 3.** The Manhattan plots reveal the p-values related to the phenotypesunder limitation of selecting participants for those carrying wild-genotype (GG)of rs2231142. The associations between SNPs and developing gout (A), betweenSNPs and developing asymptomatic hyperuricemia (AH) (B) compared to normal, andbetween gout and AH (C). The red horizontal line denotes cut-off for thep-value of significant difference between the phenotypes and variants by 1e-8.

**Additional file 9:** **SupplementaryFigure 4.** The linkage disequilibrium (LD) of between variant rs671 andrs78069066 and rs77768175 on chromosome 12 for those participants carryingrs2231142 wild-type (GG) in gene ABCG2. All the r-squares of LD between themwere greater than 0.98. The red line indicates the cut-off significant p-valueby 1e-08.

**Additional file 10:** **SupplementaryFigure 5.** The linkage disequilibrium (LD) of between variant rs10805346 andrs3733589, rs1014290 and rsrs3775948 in gene SLC2A9 which were significantlyassociated with hyperuricemia for those participants carrying rs2231142wild-type (GG; ABCG2). All the r-squares of LD between them were greater than0.22. The red line indicates the cut-off significant p-value by 1e-8.

## Data Availability

Data is not publicly available. Raw data used for this study is available from the Taiwan Biobank and China Medical University Hospital.

## Abbreviations

95% CI: 95% confidence intervals
ABCG2: ATP-binding cassette, subfamily G, member 2
ACAD10: Acyl-CoA dehydrogenase family, member 10
ADC: Arginine decarboxylase
AGMAT: Agmatinase
AH: Asymptomatic hyperuricemia
ALDH2: Aldehyde dehydrogenase 2 family
ASH1L: Ash1-like histone lysine methyltransferase
BCAS3: Breast carcinoma amplified sequence 3
BCRP1: Breast cancer resistance protein 1
BRAP: BRCA1-associated protein
CCDC63: Coiled-coil domain-containing protein 63
cGKII: Protein kinase, cGMP-dependent, type II
TLR2: Toll-like receptor 2
CLNK: Cytokine-dependent hematopoietic cell linker
CUX2: CUT-like homeobox 2
DNAJC16: DnaJ heat shock protein family member C16
GCKR: Glucokinase regulator
GLUT: Glucose transporter family
GWAS: Genome-wide association study
HECTD4: HECT domain E3 ubiquitin protein ligase 4
IL-10: Interleukin-10
LD: Linkage-disequilibrium
MAF: Minor allele frequency
MAPKAPK5: MAPK activated protein kinase 5
MEPE: Matrix extracellular phosphoglycoprotein
MSU: Monosodium urate
MTX1: Metaxin 1
MUC1: Mucin 1
NO: Nitric oxide
NOS: Nitric oxide synthase
NUDT17: Nudix hydrolase 17
OR: Odds ratios
PDZK1: PDZ domain-containing 1
PKD2: Polycystic 2
PRS: Polygenic risk score
Q4: Fourth quartile
SLC17A1: Solute carrier family 17 member 1
SLC17A3: Solute carrier family 17 member 3
SLC2A9: Solute carrier family 2 member 9
SNP: Single Nucleotide polymorphism
SPP1: Secreted phosphoprotein 1
TGF: Transforming growth factor
TRIM46: Tripartite motif-containing 46
ZNF518B: Zinc finger protein 518B
