# Supplementary material for: Polygenic risk score trend and new variants on chromosome 1 are associated with male gout in genome-wide association study
Source: Arthritis Res Ther. 2022 Oct 11;24:229. doi: 10.1186/s13075-022-02917-4 (PMC9552457; doi:10.1186/s13075-022-02917-4)
Supplement: Supplementary file 1 — Additional file 1: Supplementary Table1. The distribution of age and uric acid of those participated in the studycohort. [file 13075_2022_2917_MOESM1_ESM.docx]

Supplementary Table 1 The distribution of age and uric acid of those participated in the study cohort.

| Cohorts | Gout | Hyperuricemia | Normal | All |
| --- | --- | --- | --- | --- |
| **Discovery cohort** |  |  |  |  |
| n | 5857 | 12382 | 21355 | 39594 |
| Age (mean ± SD; years) | 54.00 ± 10.65 | 51.27 ± 11.30 | 51.72 ± 11.04 | 51.91 ± 11.10 |
| Uric acid (mean ± SD; mg/dl) | 8.79 ± 2.26 | 8.09 ± 1.12 | 5.72 ± 0.86 | 6.92 ± 1.81 |
|  |  |  |  |  |
| Base cohort |  |  |  |  |
| n | 2539 | 5649 | 13626 | 21814 |
| Age (mean ± SD; years) | 52.94 ± 10.47 | 48.60 ± 11.30 | 50.68 ± 11.10 | 50.40 ± 11.15 |
| Uric acid (mean ± SD; mg/dl) | 7.59 ± 1.76 | 7.83 ± 0.78 | 5.71 ± 0.84 | 6.48 ± 1.39 |
| Target cohort |  |  |  |  |
| n | 3318 | 6733 | 7729 | 17780 |
| Age (mean ± SD; years) | 54.68 ± 10.74 | 53.47 ± 10.81 | 53.56 ± 10.69 | 53.74 ± 10.76 |
| Uric acid (mean ± SD; mg/dl) | 9.73 ± 2.16 | 8.31 ± 1.30 | 5.74 ± 0.91 | 7.48 ± 2.11 |
| **Replication cohort** |  |  |  |  |
| n | 205 | 213 | 473 | 891 |
| Age (mean ± SD; years) | 52.12 ± 10.75 | 46.82 ± 11.24 | 50.90 ± 11.42 | 50.20 ± 11.39 |
| Uric acid (mean ± SD; mg/dl) | 9.25 ± 2.40 | 7.90 ± 0.97 | 5.69 ± 0.82 | 7.04 ± 2.05 |

p-value was estimated by ANOVA test among the three groups.
