# Supplementary material for: Polygenic risk score trend and new variants on chromosome 1 are associated with male gout in genome-wide association study
Source: Arthritis Res Ther. 2022 Oct 11;24:229. doi: 10.1186/s13075-022-02917-4 (PMC9552457; doi:10.1186/s13075-022-02917-4)
Supplement: Supplementary file 2 — Additional file 2: Supplementary Table 2. The susceptible variants significantlyassociated with gout compared to normal. [file 13075_2022_2917_MOESM2_ESM.docx]

Supplementary Table 2 The susceptible variants significantly associated with gout compared to normal.

| No. | SNP | chr | position | ref | alt | gene | p-values |
| --- | --- | --- | --- | --- | --- | --- | --- |
| 1 | rs7546668 | 1 | 15528628 | G | C | DNAJC16 | 1.01e-11 |
| 2 | rs12124078 | 1 | 15543404 | A | G | DNAJC16 | 1.47e-11 |
| 3 | rs7515244 | 1 | 15546891 | A | G | DNAJC16 | 3.40e-11 |
| 4 | rs10927807 | 1 | 15584515 | T | G | AGMAT | 4.74e-12 |
| 5 | rs12129861 | 1 | 145709377 | C | T | PDZK1 | 4.45e-09 |
| 6 | rs1471633 | 1 | 145711327 | T | G | PDZK1 | 7.81e-12 |
| 7 | rs1967017 | 1 | 145711421 | A | G | PDZK1 | 4.28e-12 |
| 8 | rs35880546 | 1 | 145722014 | A | G | CD160 | 6.43e-09 |
| 9 | rs4970874 | 1 | 145725691 | A | G | CD160 | 9.81e-09 |
| 10 | rs1471630 | 1 | 145727789 | T | C | CD160 | 9.21e-09 |
| 11 | rs9286836 | 1 | 145846532 | A | C,G | NUDT17 | 3.23e-10 |
| 12 | rs11264341 | 1 | 155179017 | C | T | TRIM46 | 7.33e-11 |
| 13 | rs4971100 | 1 | 155183255 | G | A | TRIM46 | 4.55e-11 |
| 14 | rs4072037 | 1 | 155192276 | C | T | MUC1 | 6.70e-10 |
| 15 | rs2974935 | 1 | 155212052 | G | T | MTX1 | 2.27e-09 |
| 16 | rs80142782 | 1 | 155515236 | T | C | ASH1L | 1.88e-10 |
| 17 | rs1260326 | 2 | 27508073 | T | C | GCKR | 1.46e-13 |
| 18 | rs3817588 | 2 | 27508345 | T | C | GCKR | 2.00e-11 |
| 19 | rs780094 | 2 | 27518370 | T | C | GCKR | 7.04e-12 |
| 20 | rs780093 | 2 | 27519736 | T | C | GCKR | 3.17e-12 |
| 21 | rs780092 | 2 | 27520287 | A | G | GCKR | 1.20e-10 |
| 22 | rs814295 | 2 | 27520348 | A | G | GCKR | 8.97e-11 |
| 23 | rs1260333 | 2 | 27525757 | A | G | GCKR | 3.57e-12 |
| 24 | rs73225835 | 4 | 9888911 | C | T | SLC2A9 | 1.38e-16 |
| 25 | rs10805346 | 4 | 9918723 | T | C | SLC2A9 | 3.95e-33 |
| 26 | rs3733591 | 4 | 9920506 | C | T | SLC2A9 | 2.10e-25 |
| 27 | rs13129697 | 4 | 9925343 | T | G | SLC2A9 | 1.26e-18 |
| 28 | rs6843873 | 4 | 9957164 | A | C | SLC2A9 | 1.08e-11 |
| 29 | rs3733589 | 4 | 9985700 | G | A | SLC2A9 | 1.42e-39 |
| 30 | rs3775948 | 4 | 9993558 | G | C | SLC2A9 | 1.99e-46 |
| 31 | rs1014290 | 4 | 10000237 | G | A | SLC2A9 | 5.55e-43 |
| 32 | rs6833878 | 4 | 10003931 | A | T | SLC2A9 | 5.23e-22 |
| 33 | rs117433262 | 4 | 10007547 | G | A | SLC2A9 | 5.57e-09 |
| 34 | rs1122966 | 4 | 10012852 | G | A | SLC2A9 | 2.84e-22 |
| 35 | rs733175 | 4 | 10048517 | C | T | SLC2A9 | 1.31e-12 |
| 36 | rs6834555 | 4 | 10060702 | G | A | SLC2A9 | 7.16e-13 |
| 37 | rs4697926 | 4 | 10122943 | A | C | ZNF518B | 1.80e-27 |
| 38 | rs6835689 | 4 | 10170855 | C | T | ZNF518B | 1.11e-21 |
| 39 | rs16894579 | 4 | 10176593 | C | T | ZNF518B | 2.78e-15 |
| 40 | rs17407555 | 4 | 10273370 | A | G | ZNF518B | 3.55e-32 |
| 41 | rs2192093 | 4 | 10311482 | C | T | ZNF518B | 3.61e-24 |
| 42 | rs2192090 | 4 | 10333900 | C | T | ZNF518B | 3.83e-19 |
| 43 | rs7677806 | 4 | 10381381 | C | T | ZNF518B | 1.03e-14 |
| 44 | rs10016022 | 4 | 10445282 | A | G | ZNF518B | 8.31e-09 |
| 45 | rs57755073 | 4 | 10481124 | C | A | CLNK | 2.01e-19 |
| 46 | rs7698623 | 4 | 87834676 | T | C | MEPE | 5.51e-25 |
| 47 | rs6532023 | 4 | 87852697 | T | G | MEPE | 1.86e-11 |
| 48 | rs1471403 | 4 | 87854091 | T | C | MEPE | 1.75e-11 |
| 49 | rs3061142 | 4 | 87915617 | - | ACAG | MEPE | 5.47e-27 |
| 50 | rs7675842 | 4 | 87947465 | C | T | MEPE | 2.23e-11 |
| 51 | rs72871581 | 4 | 87994628 | G | A | SPP1 | 4.22e-58 |
| 52 | rs57106923 | 4 | 88000910 | - | G | PKD2 | 5.14e-59 |
| 53 | rs2725220 | 4 | 88038770 | G | C | PKD2 | 1.00e-145 |
| 54 | rs2728104 | 4 | 88051854 | T | C | PKD2 | 3.14e-130 |
| 55 | rs2728099 | 4 | 88054586 | T | C | PKD2 | 8.34e-154 |
| 56 | rs2728125 | 4 | 88080741 | A | G | PKD2 | 3.78e-161 |
| 57 | rs2728124 | 4 | 88085008 | T | A | PKD2 | 2.37e-157 |
| 58 | rs2231164 | 4 | 88094705 | C | T | ABCG2 | 1.30e-97 |
| 59 | rs4148157 | 4 | 88099782 | G | A | ABCG2 | 1.31e-178 |
| 60 | rs2231148 | 4 | 88107326 | T | A | ABCG2 | 7.47e-61 |
| 61 | rs2054576 | 4 | 88107623 | A | G | ABCG2 | 1.64e-177 |
| 62 | rs2622621 | 4 | 88109768 | C | G | ABCG2 | 3.63e-117 |
| 63 | rs1481012 | 4 | 88117930 | A | G | ABCG2 | 4.27e-266 |
| 64 | rs2725256 | 4 | 88129846 | A | G | ABCG2 | 3.99e-33 |
| 65 | rs2231142 | 4 | 88131171 | G | T | ABCG2 | 3.17e-284 |
| 66 | rs4148155 | 4 | 88133515 | A | G | ABCG2 | 3.05e-284 |
| 67 | rs17731538 | 4 | 88134227 | G | A | ABCG2 | 3.66e-10 |
| 68 | rs4148152 | 4 | 88139757 | T | C | ABCG2 | 4.51e-84 |
| 69 | rs3114018 | 4 | 88143429 | A | C | ABCG2 | 1.80e-105 |
| 70 | rs3109823 | 4 | 88143450 | C | T | ABCG2 | 2.31e-81 |
| 71 | rs17731799 | 4 | 88147303 | G | T | ABCG2 | 2.37e-88 |
| 72 | rs2622604 | 4 | 88157772 | T | C | ABCG2 | 1.90e-75 |
| 73 | rs3114020 | 4 | 88162514 | T | C | ABCG2 | 1.00e-94 |
| 74 | rs11732936 | 4 | 88169463 | A | G | ABCG2 | 1.06e-81 |
| 75 | rs10011796 | 4 | 88169725 | T | C | ABCG2 | 1.46e-79 |
| 76 | rs7658584 | 4 | 88175489 | G | A | ABCG2 | 1.42e-12 |
| 77 | rs6815336 | 4 | 88175646 | C | T | ABCG2 | 1.82e-56 |
| 78 | rs6532055 | 4 | 88197235 | T | C | ABCG2 | 3.22e-55 |
| 79 | rs12511059 | 4 | 88205041 | C | T | ABCG2 | 2.68e-72 |
| 80 | rs72554040 | 4 | 88231172 | G | A | ABCG2 | 1.24e-71 |
| 81 | rs11729997 | 4 | 88249907 | C | A | ABCG2 | 8.57e-09 |
| 82 | rs9637599 | 4 | 88285078 | A | C | PPM1K-DT | 1.98e-15 |
| 83 | rs2869929 | 4 | 88322855 | C | T | PPM1K-DT | 1.12e-11 |
| 84 | rs11754288 | 6 | 25776721 | G | A | SLC17A4 | 2.10e-14 |
| 85 | rs9393670 | 6 | 25788833 | A | G | SLC17A1 | 1.42e-10 |
| 86 | rs2762353 | 6 | 25794203 | A | G | SLC17A1 | 2.24e-14 |
| 87 | rs1165209 | 6 | 25801091 | G | A | SLC17A1 | 4.11e-14 |
| 88 | rs1165196 | 6 | 25812922 | G | A | SLC17A1 | 1.79e-14 |
| 89 | rs1185567 | 6 | 25818360 | A | G | SLC17A1 | 2.20e-14 |
| 90 | rs1165152 | 6 | 25818538 | A | G | SLC17A1 | 4.06e-14 |
| 91 | rs765285 | 6 | 25828014 | G | C | SLC17A1 | 4.33e-14 |
| 92 | rs1165176 | 6 | 25830070 | A | G | SLC17A1 | 4.39e-14 |
| 93 | rs1185569 | 6 | 25831375 | A | G | SLC17A1 | 5.83e-14 |
| 94 | rs6905614 | 6 | 25840257 | C | A | SLC17A3 | 5.33e-14 |
| 95 | rs1408273 | 6 | 25840718 | A | G | SLC17A3 | 1.06e-13 |
| 96 | rs9393672 | 6 | 25842377 | T | G | SLC17A3 | 1.47e-13 |
| 97 | rs942379 | 6 | 25849392 | A | G | SLC17A3 | 1.92e-10 |
| 98 | rs1165160 | 6 | 25864228 | A | G | SLC17A3 | 3.78e-10 |
| 99 | rs1165207 | 6 | 25865038 | T | C | SLC17A3 | 3.34e-10 |
| 100 | rs1165205 | 6 | 25870314 | T | A | SLC17A3 | 3.63e-10 |
| 101 | rs566530 | 6 | 25878133 | T | C | SLC17A3 | 1.78e-09 |
| 102 | rs10849915 | 12 | 110895818 | T | C | CCDC63 | 1.03e-09 |
| 103 | rs10774610 | 12 | 110902439 | T | C | CCDC63 | 5.68e-09 |
| 104 | rs3782889 | 12 | 110912851 | A | G | MYL2 | 1.74e-09 |
| 105 | rs2188380 | 12 | 110948323 | T | C | LINC01405 | 1.61e-13 |
| 106 | rs12229654 | 12 | 110976657 | T | G | CUX2 | 2.97e-13 |
| 107 | rs4766566 | 12 | 111269073 | C | T | CUX2 | 1.78e-09 |
| 108 | rs3782886 | 12 | 111672685 | T | C | BRAP | 1.36e-25 |
| 109 | rs11066015 | 12 | 111730205 | G | A | ACAD10 | 1.01e-25 |
| 110 | rs4646776 | 12 | 111792215 | G | C | ALDH2 | 1.15e-25 |
| 111 | rs671 | 12 | 111803962 | G | A | ALDH2 | 4.85e-26 |
| 112 | rs78069066 | 12 | 111900120 | G | A | MAPKAPK5 | 8.39e-26 |
| 113 | rs78981331 | 12 | 112095970 | C | T | NAA25 | 7.24e-10 |
| 114 | rs2074356 | 12 | 112207597 | G | A | HECTD4 | 2.01e-12 |
| 115 | rs77768175 | 12 | 112298314 | A | G | HECTD4 | 2.69e-26 |
| 116 | rs11066280 | 12 | 112379979 | T | A | HECTD4 | 3.07e-22 |
| 117 | rs11066359 | 12 | 112607850 | C | T | RPH3A | 3.15e-13 |
| 118 | rs2079742 | 17 | 61388336 | T | C | BCAS3 | 2.09e-15 |
| 119 | rs2240736 | 17 | 61408032 | C | T | TBX2 | 3.39e-09 |
| 120 | rs757608 | 17 | 61419916 | A | G | TBX4 | 1.94e-10 |

chr: chromosome; ref: referent allele; alt: alternative allele. The p-values were estimated by chi-square test.
