# Supplementary material for: Polygenic risk score trend and new variants on chromosome 1 are associated with male gout in genome-wide association study
Source: Arthritis Res Ther. 2022 Oct 11;24:229. doi: 10.1186/s13075-022-02917-4 (PMC9552457; doi:10.1186/s13075-022-02917-4)
Supplement: Supplementary file 3 — Additional file 3: Supplementary Table 3. The susceptible variants significantly associated with hyperuricemia comparedto normal. [file 13075_2022_2917_MOESM3_ESM.docx]

Supplementary Table 3 The susceptible variants significantly associated with hyperuricemia compared to normal.

| No. | SNP | chr | position | ref | alt | gene | p-values |
| --- | --- | --- | --- | --- | --- | --- | --- |
| 1 | rs73225835 | 4 | 9888911 | C | T | SLC2A9 | 9.34e-09 |
| 2 | rs10805346 | 4 | 9918723 | T | C | SLC2A9 | 1.83e-24 |
| 3 | rs3733591 | 4 | 9920506 | C | T | SLC2A9 | 2.02e-19 |
| 4 | rs13129697 | 4 | 9925343 | T | G | SLC2A9 | 1.13e-16 |
| 5 | rs3733589 | 4 | 9985700 | G | A | SLC2A9 | 3.38e-28 |
| 6 | rs3775948 | 4 | 9993558 | G | C | SLC2A9 | 4.41e-35 |
| 7 | rs1014290 | 4 | 10000237 | G | A | SLC2A9 | 8.10e-33 |
| 8 | rs6833878 | 4 | 10003931 | A | T | SLC2A9 | 9.75e-10 |
| 9 | rs1122966 | 4 | 10012852 | G | A | SLC2A9 | 7.19e-10 |
| 10 | rs733175 | 4 | 10048517 | C | T | SLC2A9 | 1.27e-12 |
| 11 | rs6834555 | 4 | 10060702 | G | A | SLC2A9 | 1.75e-12 |
| 12 | rs4697926 | 4 | 10122943 | A | C | ZNF518B | 1.01e-11 |
| 13 | rs6835689 | 4 | 10170855 | C | T | ZNF518B | 1.82e-11 |
| 14 | rs16894579 | 4 | 10176593 | C | T | ZNF518B | 7.44e-13 |
| 15 | rs17407555 | 4 | 10273370 | A | G | ZNF518B | 9.99e-16 |
| 16 | rs2192093 | 4 | 10311482 | C | T | ZNF518B | 3.42e-18 |
| 17 | rs2192090 | 4 | 10333900 | C | T | ZNF518B | 3.25e-15 |
| 18 | rs7677806 | 4 | 10381381 | C | T | ZNF518B | 3.11e-13 |
| 19 | rs57755073 | 4 | 10481124 | C | A | CLNK | 1.10e-11 |
| 20 | rs7698623 | 4 | 87834676 | T | C | MEPE | 1.32e-11 |
| 21 | rs3061142 | 4 | 87915617 | - | ACAG | MEPE | 1.23e-11 |
| 22 | rs72871581 | 4 | 87994628 | G | A | SPP1 | 1.90e-21 |
| 23 | rs57106923 | 4 | 88000910 | - | G | PKD2 | 3.33e-21 |
| 24 | rs2725220 | 4 | 88038770 | G | C | PKD2 | 3.77e-58 |
| 25 | rs2728104 | 4 | 88051854 | T | C | PKD2 | 7.57e-55 |
| 26 | rs2728099 | 4 | 88054586 | T | C | PKD2 | 3.33e-61 |
| 27 | rs2728125 | 4 | 88080741 | A | G | PKD2 | 1.57e-61 |
| 28 | rs2728124 | 4 | 88085008 | T | A | PKD2 | 3.07e-60 |
| 29 | rs2231164 | 4 | 88094705 | C | T | ABCG2 | 4.62e-43 |
| 30 | rs4148157 | 4 | 88099782 | G | A | ABCG2 | 7.49e-71 |
| 31 | rs2231148 | 4 | 88107326 | T | A | ABCG2 | 5.00e-27 |
| 32 | rs2054576 | 4 | 88107623 | A | G | ABCG2 | 3.12e-69 |
| 33 | rs2622621 | 4 | 88109768 | C | G | ABCG2 | 1.66e-46 |
| 34 | rs1481012 | 4 | 88117930 | A | G | ABCG2 | 1.46e-103 |
| 35 | rs2725256 | 4 | 88129846 | A | G | ABCG2 | 3.63e-10 |
| 36 | rs2231142 | 4 | 88131171 | G | T | ABCG2 | 5.78e-110 |
| 37 | rs4148155 | 4 | 88133515 | A | G | ABCG2 | 4.84e-109 |
| 38 | rs4148152 | 4 | 88139757 | T | C | ABCG2 | 1.47e-33 |
| 39 | rs3114018 | 4 | 88143429 | A | C | ABCG2 | 2.16e-46 |
| 40 | rs3109823 | 4 | 88143450 | C | T | ABCG2 | 4.76e-39 |
| 41 | rs17731799 | 4 | 88147303 | G | T | ABCG2 | 1.52e-47 |
| 42 | rs2622604 | 4 | 88157772 | T | C | ABCG2 | 4.05e-42 |
| 43 | rs3114020 | 4 | 88162514 | T | C | ABCG2 | 1.38e-52 |
| 44 | rs11732936 | 4 | 88169463 | A | G | ABCG2 | 1.00e-48 |
| 45 | rs10011796 | 4 | 88169725 | T | C | ABCG2 | 1.90e-48 |
| 46 | rs6815336 | 4 | 88175646 | C | T | ABCG2 | 4.14e-37 |
| 47 | rs6532055 | 4 | 88197235 | T | C | ABCG2 | 7.05e-25 |
| 48 | rs12511059 | 4 | 88205041 | C | T | ABCG2 | 1.58e-35 |
| 49 | rs72554040 | 4 | 88231172 | G | A | ABCG2 | 3.65e-38 |
| 50 | rs3782886 | 12 | 111672685 | T | C | BRAP | 4.24e-11 |
| 51 | rs11066015 | 12 | 111730205 | G | A | ACAD10 | 1.80e-11 |
| 52 | rs4646776 | 12 | 111792215 | G | C | ALDH2 | 2.19e-11 |
| 53 | rs671 | 12 | 111803962 | G | A | ALDH2 | 1.47e-11 |
| 54 | rs78069066 | 12 | 111900120 | G | A | MAPKAPK5 | 7.55e-11 |
| 55 | rs77768175 | 12 | 112298314 | A | G | HECTD4 | 4.56e-11 |
| 56 | rs11066280 | 12 | 112379979 | T | A | HECTD4 | 3.87e-09 |
| 57 | rs2079742 | 17 | 61388336 | T | C | BCAS3 | 7.59e-09 |

chr: chromosome; ref: referent allele; alt: alternative allele; The p-values were estimated by chi-square test.
