# Supplementary material for: Polygenic risk score trend and new variants on chromosome 1 are associated with male gout in genome-wide association study
Source: Arthritis Res Ther. 2022 Oct 11;24:229. doi: 10.1186/s13075-022-02917-4 (PMC9552457; doi:10.1186/s13075-022-02917-4)
Supplement: Supplementary file 4 — Additional file 4: Supplementary Table 4. The susceptible variants significantly associated with gout compared tohyperuricemia. [file 13075_2022_2917_MOESM4_ESM.docx]

Supplementary Table 4 The susceptible variants significantly associated with gout compared to hyperuricemia.

| No. | SNP | chr | position | ref | alt | gene | p-values |
| --- | --- | --- | --- | --- | --- | --- | --- |
| 1 | rs72871581 | 4 | 87994628 | G | A | SPP1 | 2.15e-15 |
| 2 | rs57106923 | 4 | 88000910 | - | G | PKD2 | 5.96e-16 |
| 3 | rs2725220 | 4 | 88038770 | G | C | PKD2 | 1.69e-31 |
| 4 | rs2728104 | 4 | 88051854 | T | C | PKD2 | 3.39e-27 |
| 5 | rs2728099 | 4 | 88054586 | T | C | PKD2 | 4.25e-33 |
| 6 | rs2728125 | 4 | 88080741 | A | G | PKD2 | 6.02e-36 |
| 7 | rs2728124 | 4 | 88085008 | T | A | PKD2 | 3.32e-35 |
| 8 | rs2231164 | 4 | 88094705 | C | T | ABCG2 | 1.53e-20 |
| 9 | rs4148157 | 4 | 88099782 | G | A | ABCG2 | 1.49e-38 |
| 10 | rs2231148 | 4 | 88107326 | T | A | ABCG2 | 3.42e-14 |
| 11 | rs2054576 | 4 | 88107623 | A | G | ABCG2 | 5.53e-39 |
| 12 | rs2622621 | 4 | 88109768 | C | G | ABCG2 | 1.34e-29 |
| 13 | rs1481012 | 4 | 88117930 | A | G | ABCG2 | 2.89e-60 |
| 14 | rs2725256 | 4 | 88129846 | A | G | ABCG2 | 7.66e-12 |
| 15 | rs2231142 | 4 | 88131171 | G | T | ABCG2 | 7.70e-66 |
| 16 | rs4148155 | 4 | 88133515 | A | G | ABCG2 | 3.18e-66 |
| 17 | rs4148152 | 4 | 88139757 | T | C | ABCG2 | 1.50e-21 |
| 18 | rs3114018 | 4 | 88143429 | A | C | ABCG2 | 6.03e-25 |
| 19 | rs3109823 | 4 | 88143450 | C | T | ABCG2 | 1.70e-18 |
| 20 | rs17731799 | 4 | 88147303 | G | T | ABCG2 | 1.35e-16 |
| 21 | rs2622604 | 4 | 88157772 | T | C | ABCG2 | 2.01e-14 |
| 22 | rs3114020 | 4 | 88162514 | T | C | ABCG2 | 5.23e-17 |
| 23 | rs11732936 | 4 | 88169463 | A | G | ABCG2 | 4.06e-13 |
| 24 | rs10011796 | 4 | 88169725 | T | C | ABCG2 | 1.78e-12 |
| 25 | rs6532055 | 4 | 88197235 | T | C | ABCG2 | 6.30e-13 |
| 26 | rs12511059 | 4 | 88205041 | C | T | ABCG2 | 5.19e-15 |
| 27 | rs72554040 | 4 | 88231172 | G | A | ABCG2 | 4.46e-13 |

chr: chromosome; ref: referent allele; alt: alternative allele; The p-values were estimated by chi-square test.
