# Supplementary material for: Polygenic risk score trend and new variants on chromosome 1 are associated with male gout in genome-wide association study
Source: Arthritis Res Ther. 2022 Oct 11;24:229. doi: 10.1186/s13075-022-02917-4 (PMC9552457; doi:10.1186/s13075-022-02917-4)
Supplement: Supplementary file 5 — Additional file 5: Supplementary Table5. The susceptible variants of significantly associated with gout andhyperuricemia in those with wild genotype of top significant variants(rs2231142). [file 13075_2022_2917_MOESM5_ESM.docx]

Supplementary Table 5 The susceptible variants of significantly associated with gout and hyperuricemia in those with wild genotype of top significant variants (rs2231142).

| No. | SNP | chr | position | ref | alt | gene | p-values |
| --- | --- | --- | --- | --- | --- | --- | --- |
| Related to gout while compared to normal under rs2231142 genotype GG (n=13088) | | | | | | | |
| 1 | rs3733589 | 4 | 9985700 | G | A | SLC2A9 | 1.83e-10 |
| 2 | rs3775948 | 4 | 9993558 | G | C | SLC2A9 | 1.84e-13 |
| 3 | rs1014290 | 4 | 10000237 | G | A | SLC2A9 | 1.26e-12 |
| 4 | rs17407555 | 4 | 10273370 | A | G | ZNF518B | 5.81e-10 |
| 5 | rs2725231 | 4 | 88013803 | A | G | PKD2 | 2.64e-09 |
| 6 | rs3109823 | 4 | 88143450 | C | T | ABCG2 | 2.68e-09 |
| 7 | rs2622604 | 4 | 88157772 | T | C | ABCG2 | 2.71e-13 |
| 8 | rs6532055 | 4 | 88197235 | T | C | ABCG2 | 9.46e-10 |
| 9 | rs72554040 | 4 | 88231172 | G | A | ABCG2 | 1.40e-10 |
| 10 | rs671 | 12 | 111803962 | G | A | ALDH2 | 6.31e-09 |
| 11 | rs78069066 | 12 | 111900120 | G | A | MAPKAPK5 | 9.49e-09 |
| 12 | rs77768175 | 12 | 112298314 | A | G | HECTD4 | 6.84e-09 |
|  |  |  |  |  |  |  |  |
| Related to hyperuricemia compared to normal under rs2231142 genotype GG (n=16395) | | | | | | | |
| 1 | rs10805346 | 4 | 9918723 | T | C | SLC2A9 | 5.74e-09 |
| 2 | rs3733589 | 4 | 9985700 | G | A | SLC2A9 | 7.13e-12 |
| 3 | rs3775948 | 4 | 9993558 | G | C | SLC2A9 | 5.17e-17 |
| 4 | rs1014290 | 4 | 10000237 | G | A | SLC2A9 | 5.77e-15 |

chr: chromosome; ref: referent allele; alt: alternative allele; The p-values were estimated by chi-square test.
