# Supplementary material for: Polygenic risk score trend and new variants on chromosome 1 are associated with male gout in genome-wide association study
Source: Arthritis Res Ther. 2022 Oct 11;24:229. doi: 10.1186/s13075-022-02917-4 (PMC9552457; doi:10.1186/s13075-022-02917-4)
Supplement: Supplementary file 6 — Additional file 6: SupplementaryFigure 1. The linkage disequilibrium of the four variants significantlyassociated with gout in genes DNAJC16 and AGMAT. The variant rs7546668 showedhigh associations with the other three variants (r2 >=0.68). Thered line indicates the cut-off significant p-value by 1e-8. [file 13075_2022_2917_MOESM6_ESM.docx]

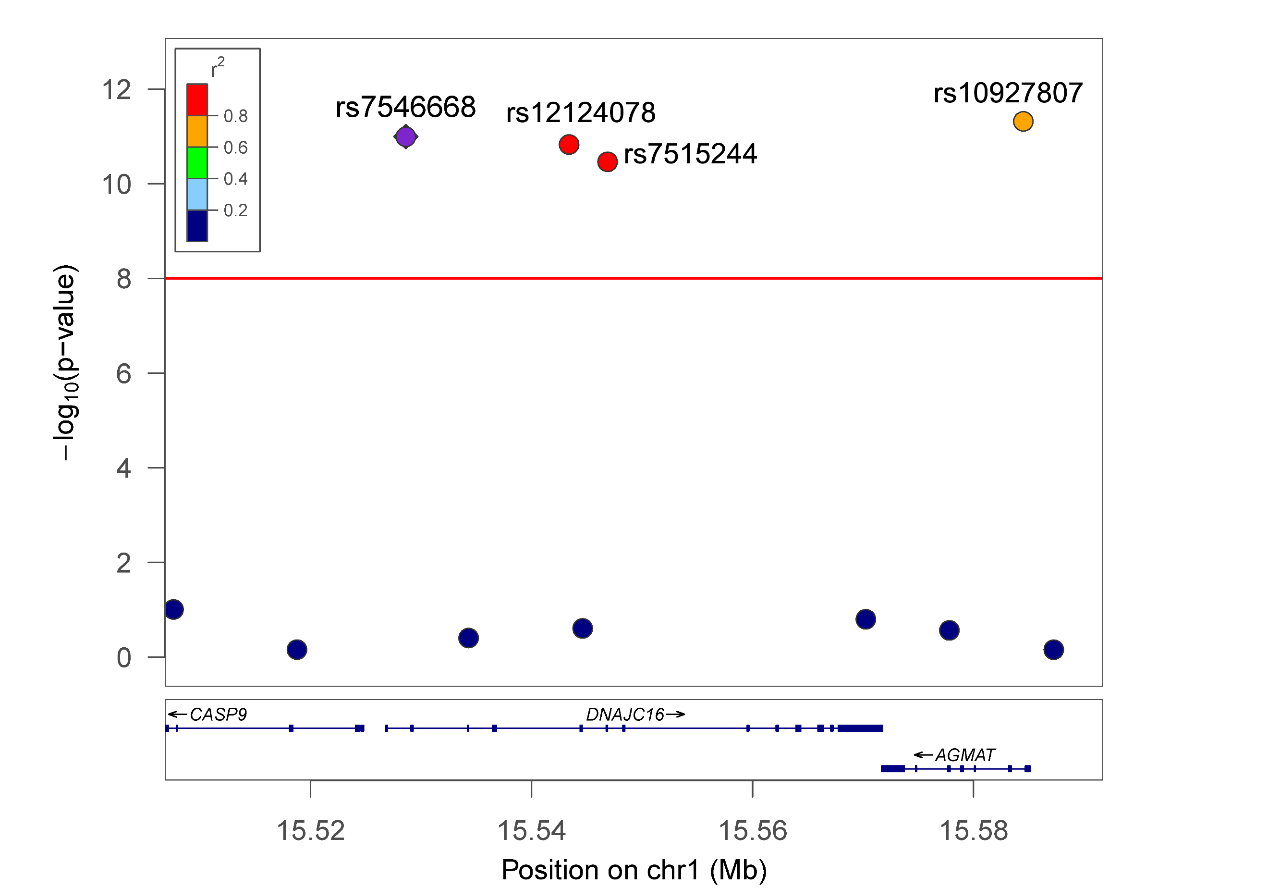


Supplementary Figure 1. The linkage disequilibrium of the four variants significantly associated with gout in genes DNAJC16 and AGMAT. The variant rs7546668 showed high associations with the other three variants (r^2^ >=0.68). The red line indicates the cut-off significant p-value by 1e-8.
