# Supplementary material for: Polygenic risk score trend and new variants on chromosome 1 are associated with male gout in genome-wide association study
Source: Arthritis Res Ther. 2022 Oct 11;24:229. doi: 10.1186/s13075-022-02917-4 (PMC9552457; doi:10.1186/s13075-022-02917-4)
Supplement: Supplementary file 7 — Additional file 7: Supplementary Figure 2. The paired linkagedisequilibrium among the variants which were significantly associated withhyperuricemia on chromosome 4 (A) and 12 (B). [file 13075_2022_2917_MOESM7_ESM.docx]

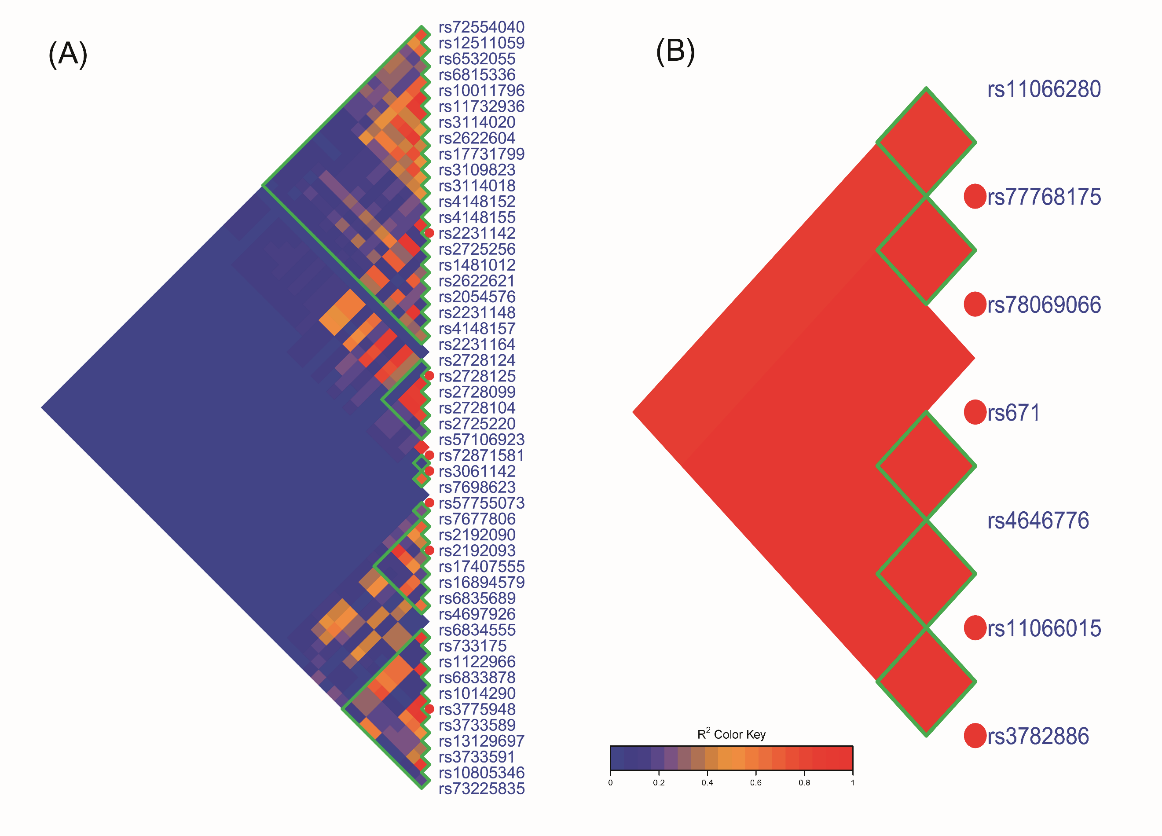


Supplementary Figure 2. The paired linkage disequilibrium among the variants which were significantly associated with hyperuricemia on chromosome 4 (A) and 12 (B).
