# Supplementary material for: Polygenic risk score trend and new variants on chromosome 1 are associated with male gout in genome-wide association study
Source: Arthritis Res Ther. 2022 Oct 11;24:229. doi: 10.1186/s13075-022-02917-4 (PMC9552457; doi:10.1186/s13075-022-02917-4)
Supplement: Supplementary file 8 — Additional file 8: SupplementaryFigure 3. The Manhattan plots reveal the p-values related to the phenotypesunder limitation of selecting participants for those carrying wild-genotype (GG)of rs2231142. The associations between SNPs and developing gout (A), betweenSNPs and developing asymptomatic hyperuricemia (AH) (B) compared to normal, andbetween gout and AH (C). The red horizontal line denotes cut-off for thep-value of significant difference between the phenotypes and variants by 1e-8. [file 13075_2022_2917_MOESM8_ESM.docx]

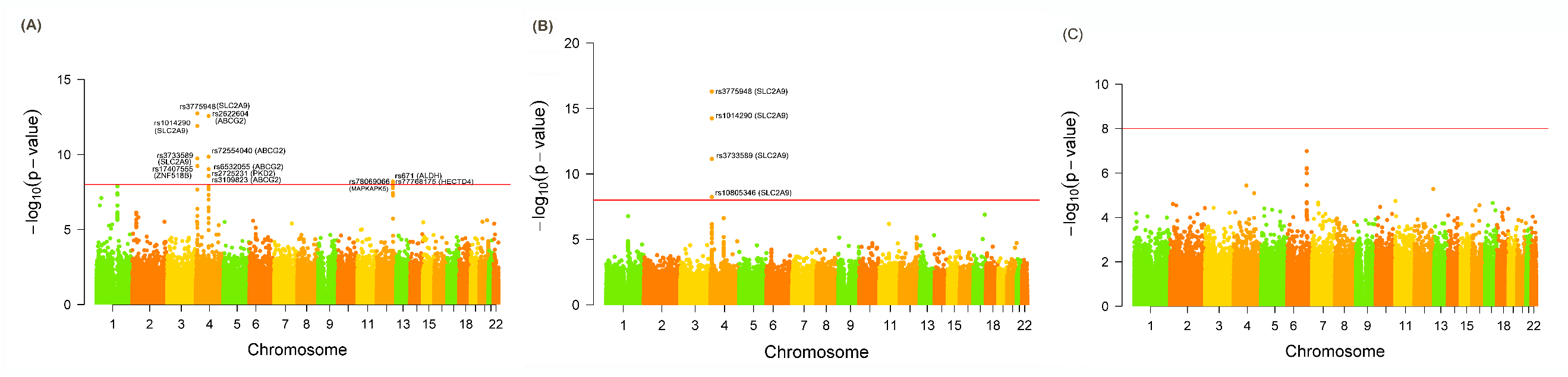


Supplementary Figure 3. The Manhattan plots reveal the p-values related to the phenotypes under limitation of selecting participants for those carrying wild-genotype (GG) of rs2231142. The associations between SNPs and developing gout (A), between SNPs and developing asymptomatic hyperuricemia (AH) (B) compared to normal, and between gout and AH (C). The red horizontal line denotes cut-off for the p-value of significant difference between the phenotypes and variants by 1e-8.
