# Supplementary material for: Polygenic risk score trend and new variants on chromosome 1 are associated with male gout in genome-wide association study
Source: Arthritis Res Ther. 2022 Oct 11;24:229. doi: 10.1186/s13075-022-02917-4 (PMC9552457; doi:10.1186/s13075-022-02917-4)
Supplement: Supplementary file 9 — Additional file 9: SupplementaryFigure 4. The linkage disequilibrium (LD) of between variant rs671 andrs78069066 and rs77768175 on chromosome 12 for those participants carryingrs2231142 wild-type (GG) in gene ABCG2. All the r-squares of LD between themwere greater than 0.98. The red line indicates the cut-off significant p-valueby 1e-08. [file 13075_2022_2917_MOESM9_ESM.docx]

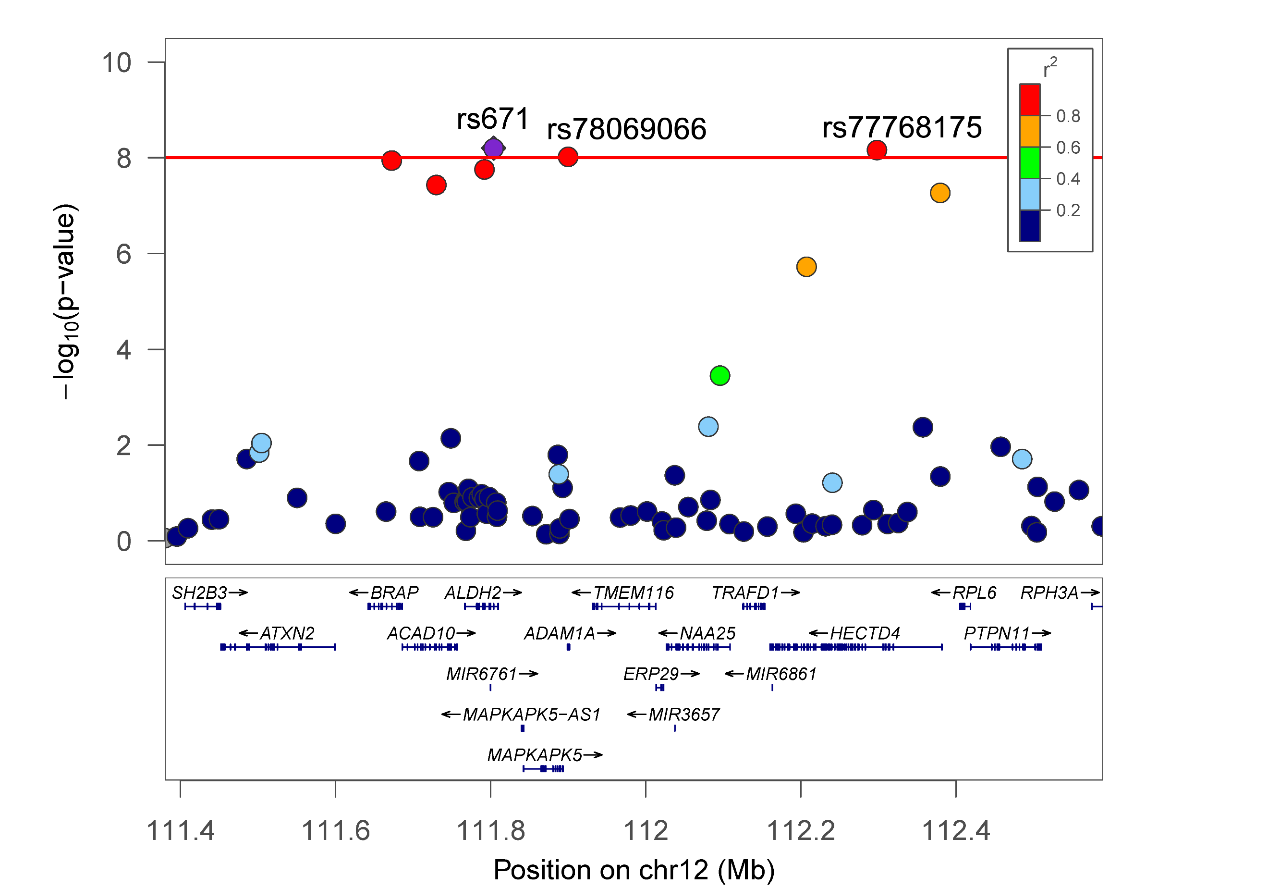


Supplementary Figure 4. The linkage disequilibrium (LD) of between variant rs671 and rs78069066 and rs77768175 on chromosome 12 for those participants carrying rs2231142 wild-type (GG) in gene ABCG2. All the r-squares of LD between them were greater than 0.98. The red line indicates the cut-off significant p-value by 1e-08.
