# Supplementary material for: Polygenic risk score trend and new variants on chromosome 1 are associated with male gout in genome-wide association study
Source: Arthritis Res Ther. 2022 Oct 11;24:229. doi: 10.1186/s13075-022-02917-4 (PMC9552457; doi:10.1186/s13075-022-02917-4)
Supplement: Supplementary file 10 — Additional file 10: SupplementaryFigure 5. The linkage disequilibrium (LD) of between variant rs10805346 andrs3733589, rs1014290 and rsrs3775948 in gene SLC2A9 which were significantlyassociated with hyperuricemia for those participants carrying rs2231142wild-type (GG; ABCG2). All the r-squares of LD between them were greater than0.22. The red line indicates the cut-off significant p-value by 1e-8. [file 13075_2022_2917_MOESM10_ESM.docx]

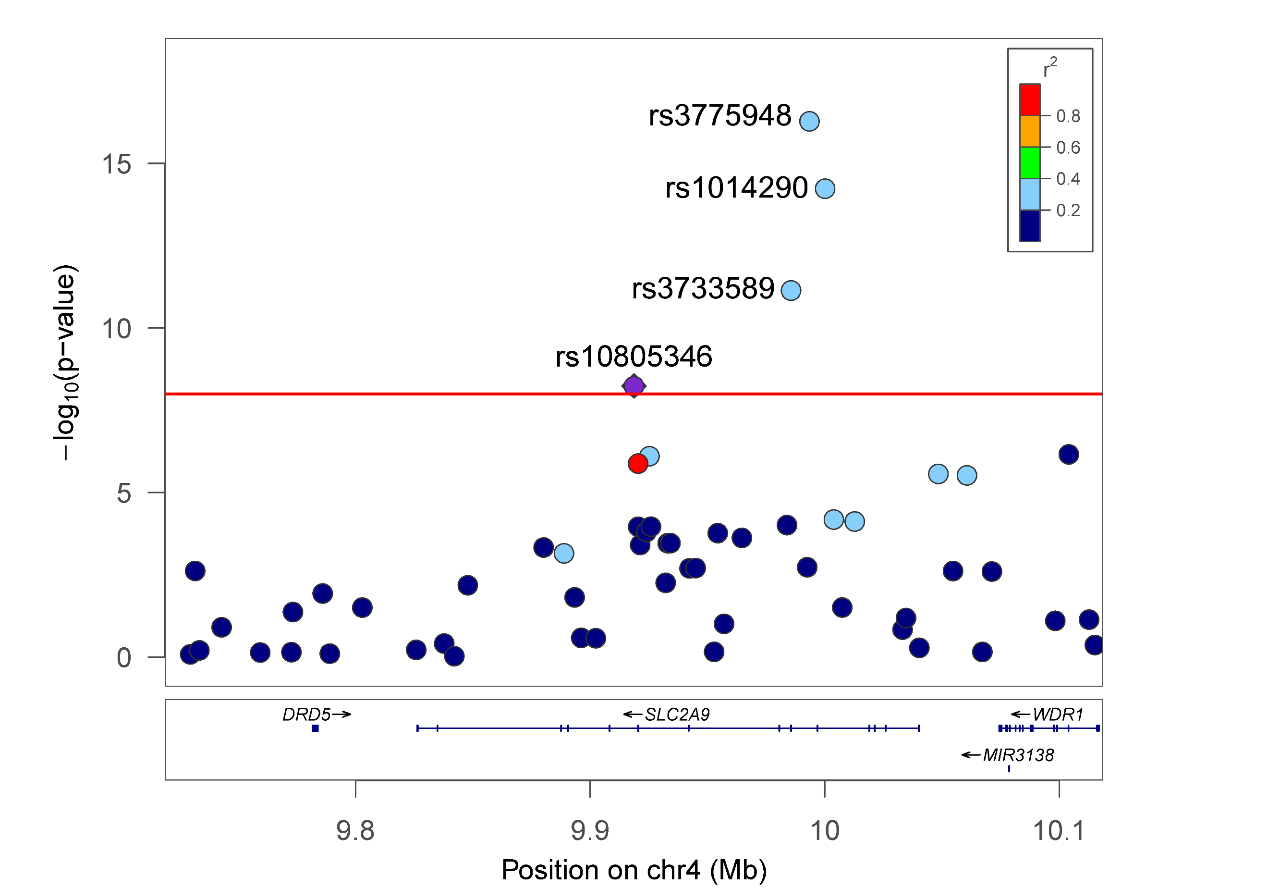


Supplementary Figure 5. The linkage disequilibrium (LD) of between variant rs10805346 and rs3733589, rs1014290 and rsrs3775948 in gene SLC2A9 which were significantly associated with hyperuricemia for those participants carrying rs2231142 wild-type (GG; ABCG2). All the r-squares of LD between them were greater than 0.22. The red line indicates the cut-off significant p-value by 1e-8.
